# Self-reported changes in physical activity, sedentary behavior, and screen time among informal caregivers during the COVID-19 pandemic

**DOI:** 10.1186/s12889-021-11294-7

**Published:** 2021-07-02

**Authors:** Mary L. Greaney, Zachary J. Kunicki, Megan M. Drohan, Christie L. Ward-Ritacco, Deborah Riebe, Steven A. Cohen

**Affiliations:** 1 Department of Health Studies, University of Rhode Island, 25 West Independent Way, Kingston, Rhode Island 02881 USA; 2 Department of Psychiatry and Human Behavior, Warren Alpert Medical School of Brown University, Providence, Rhode Island USA; 3 Department of Psychology, University of Rhode Island, Kingston, Rhode Island USA; 4 Department of Kinesiology, University of Rhode Island, Kingston, Rhode Island USA

**Keywords:** COVID-19, Informal caregivers, Physical activity, Screen time, Sedentary behavior

## Abstract

**Background:**

Informal caregivers providing unpaid assistance may be vulnerable to changes in health behaviors due to modifications in caregiving during the COVID-19 pandemic. Therefore, this cross-sectional study explored self-reported changes in physical activity (PA), sedentary behavior, and screen time among informal caregivers providing care for older adults aged 50+ during the pandemic.

**Methods:**

Study participants were recruited via Amazon’s Mechanical Turk and reported their perceived changes (increased a lot, increased a little, remained the same, decreased a little, decreased a lot) in moderate-intensity PA (MPA), vigorous-intensity PA (VPA), sedentary behavior, and screen time (weekday and weekend) during the pandemic. For analytic purposes, response categories were categorized into three-level ordinal variables—increased (increased a lot, increased a little), no change (remained the same), decreased (decreased a little, decreased a lot). Multinomial logistic regression models assessed the likelihood of changes (vs. no change) in  MPA,  VPA, sedentary behavior, and screen time (weekday, weekend) based on caregiving and demographic characteristics.

**Results:**

In total, 2574 individuals accessed the study link, 464 of whom did not meet eligibility requirements. In addition, people who completed 80% or less of the survey (*n* = 1171) and/or duplicate IP addresse (*n* = 104) were excluded, resulting in an analytic sample of *n* = 835. The sample was 69% male, had a mean age of 34 (SD = 9.7), and 48% reported increased VPA, while 55% reported increased MPA. The majority also reported increased sedentary behavior, as well as increased screen time. Respondents living with their care recipient were more likely to report increased weekday screen time (Odds Ratio [OR] = 1.55, 95% CI 1.11–2.16) and sedentary behavior (OR = 1.80, 95% CI 1.28–2.53) than respondents not living with the care recipient. Those living with their care recipient were also more likely to reported increased MPA (OR = 1.64, 95% CI 1.16–2.32), and VPA (OR = 1.53, 95% CI 1.09–2.15), but also more likely to report a decrease in VPA (OR = 1.75, 95% CI 1.14–2.70).

**Conclusion:**

The majority of respondents reported that their MPA, VPA PA, sedentary behavior, and screen time had changed during the pandemic. Living with the care recipient was associated with both positive and negative changes in behavior. Future research can explore factors associated with these reported changes in behavior.

The World Health Organization declared COVID-19 a pandemic in March 2020 [1, 2], and efforts to curtail the pandemic have included closing public venues, sheltering in place, practicing social distancing, and restricting visitors to nursing homes and long-term care facilities [3]. There is a growing body of research suggesting that the COVID-19 pandemic has negatively impacted participation in healthful behaviors, including physical activity (PA) [4–6].

An estimated 41 million people in the United States are informal caregivers, individuals who provide unpaid care or assistance to older adults, persons with disabilities, and individuals requiring assistance [7], and this number will likely grow due to the increasing prevalence of chronic diseases and population aging [8]. A study analyzing Behavioral Risk Factor Surveillance System (BRFSS) data from 44 states, Washington DC, and Puerto Rico found that between 2015 and 2017 approximately 20% of respondents had provided care for a relative or friend in the last month [8]. Being an informal caregiver has associated benefits such as feeling closer to the care recipient [9, 10] and finding purpose in being a caregiver [11]. However, research also indicates that informal caregivers may have higher levels of stress and depression, as well as lower levels of subjective well-being and physical health, compared to non-caregivers [12–14].

Research shows caregiver burden and caregiving intensity may negatively impact caregivers’ ability to engage in healthy behaviors [15], and increase caregiver risk of poor health and chronic diseases [15, 16]. For example, recent research conducted in Poland found that informal caregivers have less time for PA, hobbies, and their social lives, especially those who reside with their care recipient, compared to non-caregivers [17]. It is of public health concern that informal caregivers may be limited in their ability to engage in PA due to its positive association with mental health [18], and many other well-known benefits including decreased risk for all-cause mortality and cardiovascular disease [19], some cancers [20], depression [21], and weight loss maintenance [22]. On the other hand, prolonged periods of sedentary behavior (SB, [e.g., sitting, watching television]) have negative impacts on health [23, 24], including elevated risks of all-cause mortality [24].

There is concern that the ongoing COVID-19 pandemic may negatively impact informal caregivers’ participation in health-promoting behaviors and their health status [25], and research is needed to determine if intervention efforts are needed. Informal caregivers may be particularly vulnerable to changes in behaviors during the ongoing pandemic. Efforts to curtail the spread and impacts of COVID-19 likely resulted in changes in caregiving responsibilities for informal caregivers, including increases in caregiving responsibilities and caregiver burden and strain [25] and impacts on physical and mental health and health-related quality of life [26, 27]. Informal caregivers have reported that caregiver burden has increased during the pandemic [28, 29]. Therefore, the aim of the current exploratory study was to determine if informal caregivers for older adults perceived that their health behaviors (moderate-intensity PA [MPA], vigorous-intensity PA [VPA], SB, weekday screen time [ST], and weekend ST) had changed since the start of the pandemic. It has been hypothesized that there would be an increase in negative health behaviors (e.g., SB and ST) and a decrease in positive health behaviors (e.g., PA) due to changes in informal caregiving responsibilities that occurred as a result of efforts to curtail the spread of COVID-19.

## Methods

Study participants were recruited in June 2020 using Amazon’s Mechanical Turk (MTurk), an online platform/labor market that is used to recruit study participants globally for social and behavioral science research [30–32]. Registered MTurk users, referred to as workers, complete Human Intelligence Tasks (HITs), surveys and/or tasks, for a small incentive. Registered users log in to the MTurk online platform where they can access a list of HITs that they may be eligible to complete. The HIT for the current study was posted on the MTurk platform. Interested individuals accessing a link to Qualtrics where they accessed the informed consent document, provided informed consent, and completed questions assessing eligibility, which included being an informal caregiver for an individual 50 years or older with a health condition, disability, or cognitive decline, living in the United States, and being able to read English. Participants received $1.50 for their effort and the study was approved by the Institutional Review Board at the University of Rhode Island (study # 1606088-2).

### Measures

#### Outcome measures

The five outcome measures were self-reported changes in behaviors due to the pandemic: change in MPA, change in VPA, change in SB, change in weekday ST, and change in weekend (Saturday, Sunday) ST. Participants reported if they felt their participation in these behaviors had decreased a lot, decreased a little, remained the same, increased a little, or increased a lot since the start of the COVID-19 pandemic. Due to the novel nature of the pandemic and the lack of existing measures assessing perceived change in behavior due to the COVID-19, the outcomes measures were created for this current study. For analytic purposes, response categories were categorized into three-level ordinal variables—increased (increased a lot, increased a little), no change (remained the same), decreased (decreased a little, decreased a lot) due to small sample sizes in the “decreased a lot” categories), which would have left empty cells using multivariable regression models (MPA [*n* = 26], VPA [*n* = 37], SB [*n* = 12], weekday ST [*n* = 11], and weekend ST [*n* = 17]).

#### Covariates

Covariates included the caregiver’s gender (male, female), age, ethnicity (non-Hispanic, Hispanic), household income (<$15,000, $15,000 < $50,000, $50,000 < $75,000, $ > 75,000), and if they had ever been diagnosed with COVID-19 (yes, no). Respondents also reported their racial identify, which based on the distribution of the data was dichotomized as White and Non-White. Caregivers also completed the 12-item Caregiver Burden Index (CBI), a multidimensional scale that estimates the amount of burden caregivers experience due to their caregiving [33]. Scores ranged from 0 to 43 and higher CBI scores indicate greater caregiver burden. The CBI was reliable in this sample (ω = .90).

Respondents’ aerobic PA during the pandemic also was included as a covariate. It was assessed using the BRFSS PA module, a validated measure [34], that has been modified to be self-administered versus interviewer-administered [35, 36]. Respondents completed the assessment of their PA before reporting their perceived changes in their MPA and VPA due to COVID-19. Two items assessed MPA, with the first item asking participants to report how many days (range 0–7) in a usual week that they did moderate activities for at least 10 min at a time, such as brisk walking, bicycling, vacuuming, gardening, or anything else that causes small increases in breathing or heart rate. The second item asked respondents to report the total time spent per day on MPA (On days when you do moderate activities for at least 10 min at a time, how much total time per day do you spend doing these activities?). VPA was assessed using two similar items. Reported minutes of MPA and VPA were summed into a total number of weekly minutes and then dichotomized as to whether a person met (1 = yes, 0 = no) the current recommendation put forth by the Centers for Disease Control and Prevention of 150+ min of MPA, or 75+ min of VPA, or an equivalent combination of both per week [19].

### Analysis

The data were checked to ensure there was only one record per participant by checking IP addresses before analysis. If duplicate IP addresses were detected, only the first response was used for analysis. After selecting for covariates of interest, missing data diagnostics showed 2% of the data were missing. These data were imputed using multiple imputation with *m* = 10 imputations. Multiple imputation was used to handle the missing data since it is a currently recommended technique which performs well when imputing categorical data [37]. Descriptive statistics were calculated for all study variables, including means and standard deviations for continuous measures and frequencies for categorical variables. Five separate multinomial logistic regression models, one for each behavior, were constructed to explore the relationships between the study covariates and the outcome variables. All models included covariates and the “no change” response option was the reference category in all multinomial regression models. This means the odds ratios (OR) for increases in behavior (MPA, VPA, SB, or ST) during the pandemic represent the likelihood of increases in behavior compared to “no change” in the examined behavior. Similarly, the ORs for decreases in behavior are compared to “no change” in the assessed behavior. Analyses were conducted using R version 4.0.2 (R Core Team, 2020) using the *mice* package for imputation and *nnet* package for multinomial logistic regression [38, 39].

## Results

In total, 2574 individuals accessed the study link: 464 of whom did not meet eligibility requirements. Additionally, people who completed 80% or less of the survey (*n* = 1171) and e duplicate IP addresses (*n* = 104) were excluded, resulting in an analytic sample of *n* = 835.

The majority of the sample (69%, *n* = 572) was male, 45% (*n* = 374) identified as Hispanic, 55% (*n* = 457) identified as White, and 53% (*n* = 444) reported a COVID-19 diagnosis. Respondents average age was 34 (SD = 10) years, and 41% (*n* = 339) of respondents were living with their care recipient. See Table 1 for additional detail. With respect to health behaviors, 33% (*n* = 272) of respondents were classified as meeting the current PA recommendation for aerobic exercise.

**Table 1:** Description of Sample

Characteristic	*N or Mean*	*% or SD*
Age	33.97	9.7
Caregiver Burden Index	20.42	7.77
Lives with Care Recipient		
Yes	339	41%
No	496	59%
Met Physical Activity Guidelines		
Yes	272	33%
No	563	67%
Relationship to Care Recipient		
Adult Child	559	67%
Spouse	55	7%
Other	221	26%
Gender Identity		
Female	263	31%
Male	572	69%
Ethnicity		
Non-Hispanic	461	55%
Hispanic	374	45%
Racial Identity		
White	457	55%
Non-White	378	45%
Income		
< $15,000	131	16
$15,000 < $50,000	230	28
$50,000 < $75,000	173	21
$ > 75,000	301	36
COVID-19 Diagnosis		
Yes	444	53%
No	391	47%

Overall, 55% (*n* = 459) of respondents reported an increase in MPA, while 29% (*n* = 244) reported no change, and 16% (*n* = 132) reported a decrease. Model 1 examined changes in MPA and revealed that women were more likely than men to report an increase (vs. no change) in MPA (OR = 1.03, 95% CI: 1.01, 1.04). In addition, respondents who reported living with their care recipient (OR = 1.64, 95% CI: 1.16, 2.32) were more likely to report an increase in MPA (vs. no change) than respondents who were not living with the care recipient, as were respondents who had reported COVID-19 diagnosis (OR = 1.48, 95% CI: 1.04, 2.12). No other significant results emerged (see Table 2).

**Table 2:** Multinomial Logistic Regression of Self-Reported Changes in Caregivers' Physical Activity (PA)

	Moderate PA		Vigorous PA	
	Odds Ratios (95% Confidence Interval)		Odds Ratios (95% Confidence Interval)	
Caregiver variables	Decreased	Increased	Decreased	Increased
Gender				
Male	Ref	Ref	Ref	Ref
Female	1.10 (.70–1.74)	1.00 (.71–1.42)	1.44 (.94–2.21)	1.14 (.80–1.61)
Age	1.01 (.98–1.03)	1.03** (1.01–1.04)	1.00 (.98–1.02)	1.01 (.99–1.03)
Ethnicity				
Non-Hispanic	Ref	Ref	Ref	Ref
Hispanic	.84 (.52–1.34)	1.33 (.94–1.88)	.72 (.46–1.14)	1.33 (.95–1.87)
Race				
White	Ref	Ref	Ref	Ref
Non-White	.88 (.55–1.42)	1.31 (.92–1.85)	.78 (.50–1.22)	1.13 (.80–1.58)
Household income				
< $15,000	Ref	Ref	Ref	Ref
$15,000 - $50,000	1.11 (.54–2.30)	.82 (.49–1.38)	1.84 (.92–3.67)	1.00 (.61–1.63)
$50,001 - $75,000	1.00 (.45–2.19)	1.08 (.62–1.88)	1.35 (.65–2.82)	1.02 (.60–1.73)
$ > 75,000	1.34 (.67–2.68)	.87 (.52–1.45)	1.75 (.89–3.42)	1.17 (.72–1.90)
COVID-19 diagnosis				
No	Ref	Ref	Ref	Ref
Yes	1.07 (.66–1.72)	1.48* (1.04–2.12)	.81 (.52–1.28)	1.68** (1.18–2.38)
CBI score	.98 (.95–1.01)	.99 (.97–1.01)	1.00 (.98–1.03)	1.03* (1.00–1.05)
Met PA recommendation^a^				
No	Ref	Ref	Ref	Ref
Yes	.96 (.57–1.61)	1.36 (.94–1.96)	.86 (.52–1.41)	1.27 (.89–1.81)
Lives with care recipient				
No	Ref	Ref	Ref	Ref
Yes	1.33 (.84–2.12)	1.64^**^ (1.16–2.32)	1.75^*^ (1.14–2.70)	1.53^*^ (1.09–2.15)
Relationship to care recipient				
Adult child	Ref	Ref	Ref	Ref
Spouse	.88 (.29–2.66)	1.77 (.87–3.60)	.88 (.36–2.17)	1.03 (.54–1.96)
Other	1.20 (.73–1.99)	1.15 (.78–1.69)	1.57 (.97–2.53)	1.41 (.96–2.07)

The “no change” category was used as the reference group; ^a^150 minutes of moderate PA/week or 75 min of vigorous PA/week, or an equivalent combination of the two/week; * = *p* < .05, ** = *p* < .01

Across the sample, 48% (*n* = 403) of respondents reported an increase in VPA, while 33% (*n* = 278) reported no change, and 18% (*n* = 154) reported a decrease. Model 2 examined reported changes in VPA and determined that participants who had been diagnosed with COVID-19 (OR = 1.68, 95% CI: 1.18, 2.38), and those who reported higher levels of caregiver burden as assessed by the Caregiver Burden Index (OR = 1.03, 95% CI: 1.00, 1.05) were also more likely to report in an increase (vs. no change) in VPA. Participants who reported living with their care recipient were more likely to report increased (OR = 1.53, 95% CI: 1.09, 2.15), or decreased (OR = 1.75, 95%CI: 1.14, 2.79) VPA (vs. no change). There were no other significant associations (see Table 2).

In total, 63% (*n* = 526) of study participants reported an increase in SB, while 28% (*n* = 237) reported no change, and 16% (*n* = 132) reported a decrease. Model 3 assessed changes in SB and showed that participants who reported living with their care recipient (OR = 1.80, 95% CI: 1.28, 2.53) were more likely to report increased SB (vs. no change) compared to respondents not living with the care recipient. No other significant results emerged (see Table 3).

**Table 3:** Multinomial Logistic Regression of Self-Reported Changes in Caregivers' Sedentary Behavior

	Odds Ratios (95% Confidence Interval)	
Caregiver Variable	Decreased	Increased
Gender		
Male	Ref	Ref
Female	1.28 (.72–2.27)	1.22 (.87–1.73)
Age	.98 (.95–1.01)	1.00 (.99–1.02)
Ethnicity		
Non-Hispanic	Ref	Ref
Hispanic	.69 (.38–1.25)	1.19 (.85–1.67)
Race		
White	Ref	Ref
Non-White	1.13 (.63–2.04)	1.37 (.97–1.92)
Household income		
< $15,000	Ref	Ref
$15,000 - $50,000	1.37 (.49–3.85)	.69 (.41–1.16)
$50,001 - $75,000	1.38 (.47–4.05)	.65 (.37–1.13)
$ > 75,000	1.71 (.63–4.67)	.70 (.42–1.16)
COVID-19 diagnosis		
No	Ref	Ref
Yes	.75 (.42–1.37)	.99 (.70–1.40)
CBI score	1.04 (1.00–1.07)	1.00 (.98–1.02)
Met PA recommendation^a^		
No	Ref	Ref
Yes	1.03 (.56–1.89)	.91 (.64–1.30)
Lives with care recipient		
No	Ref	Ref
Yes	1.29 (.72–2.31)	1.80^**^ (1.28–2.53)
Relationship to care recipient		
Adult Child	Ref	Ref
Spouse	.74 (.23–2.36)	.97 (.51–1.84)
Other	.89 (.46–1.74)	1.25 (.86–1.82)

The no change category was used as the reference group;; ^a^150 minutes of moderate PA/week or 75 min of vigorous PA/week, or an equivalent combination of the two/week;. ** = *p* < .01

More than half of respondents (61%, *n* = 510) reported that their weekday ST had increased, while 30% (*n* = 253) reported no change, and 9% (*n* = 72) reported their weekday ST had decreased. Model 4 examined changes in weekday ST and found that participants who lived with their care recipient (OR = 1.55, 95% CI: 1.11, 2.16) had higher odds of increased weekday ST (vs. no change). In addition, over half (57% *n* = 453) of respondents reported that their weekend ST had increased, while 33% (n = 278) reported no change, and 10% (*n* = 80) reported a decrease. The results of Model 5 examined changes in weekend ST and revealed that participants who reported a COVID-19 diagnosis had higher odds (OR = 1.99, 95% CI: 1.13, 3.53) of reporting a decrease in weekend ST. As seen in Table 4, no other significant associations were identified for weekday or weekend ST.

**Table 4:** Multinomial Logistic Regression of Self-Reported Changes in  Caregivers’ Weekday and Weekend Screen Time

	Weekday Screen Time		Weekend Screen Time	
	Odds Ratios (95% Confidence Interval l)		Odds Ratios (95% Confidence Intervall)	
Caregiver Variables	Decreased	Increased	Decreased	Increased
Gender				
Male	Ref	Ref	Ref	Ref
Female	1.30 (.74–2.28)	1.06 (.75–1.49)	1.06 (.62–1.81)	.80 (.58–1.10)
Age	.99 (.97–1.02)	1.00 (.99–1.02)	.99 (.97–1.02)	1.00 (.98–1.01)
Ethnicity				
Non-Hispanic	Ref	Ref	Ref	Ref
Hispanic	.93 (.52–1.65)	1.23 (.88–1.72)	.82 (.47–1.42)	.92 (.66–1.27)
Race				
White	Ref	Ref	Ref	Ref
Non-White	1.12 (.62–2.01)	1.29 (.92–1.80)	.62 (.35–1.10)	1.07 (.77–1.47)
Household income				
< $15,000	Ref	Ref	Ref	Ref
$15,000 - $50,000	.77 (.31–1.92)	.63 (.38–1.03)	1.53 (.57–4.16)	.71 (.44–1.14)
$50,001 - $75,000	1.00 (.37–2.67)	.87 (.51–1.49)	1.87 (.66–5.31)	1.00 (.59–1.67)
$ > 75,000	1.71 (.71–4.11)	.98 (.60–1.62)	2.52 (.96–6.61)	.96 (.60–1.53)
COVID-19 diagnosis		Ref	Ref	
No	Ref	Ref	1.53 (.57–4.16)	.71 (.44–1.14)
Yes	0.99 (.54–1.80)	.83 (.59–1.12)	1.87 (.66–5.31)	1.00 (.59–1.67)
CBI score	1.01 (.97–1.05)	.98 (.96–1.00)	2.52 (.96–6.61)	.96 (.60–1.53)
Met PA recommendation ^a^				
No	Ref	Ref	Ref	Ref
Yes	1.43 (.80–2.55)	.88 (.62–1.25)	.58 (.32–1.07)	1.03 (.73–1.44)
Lives with care recipient				
No	Ref	Ref	Ref	Ref
Yes	.93 (.51–1.68)	1.55* (1.11–2.16)	1.10 (.64–1.89)	1.28 (.93–1.77)
Relationship to care recipient				
Adult child	Ref	Ref	Ref	Ref
Spouse	2.04 (.74–5.58)	1.68 (.84–3.36)	1.59 (.60–4.25)	1.41 (.73–2.72)
Other	1.02 (.53–1.96)	1.29 (.89–1.88)	.83 (.44–1.57)	.95 (.66–1.36)

The “no change” category was used as the reference group; ^a^150 minutes of moderate PA/week or 75 min of vigorous PA/week, or an equivalent combination of the two/week; * = *p* < .05

## Discussion

A positive finding of the current study was that 48% of respondents reported that their VPA increased and 55% reported increased MPA. Similarly, although different PA measures were used, a study with adults (80% White, 15% with a COVID-19 diagnosis) from the United Kingdom (UK) found that 47% of participants reported exercising more during the social lockdown, while 35% stated that they were exercising less [40]. Another UK study found that a smaller percentage of respondents (11%) reported being more physically active, while 25% reported doing less PA during lockdown [41]. Neither of these studies assessed caregiving status. Other studies also report that PA participation has declined during the pandemic [42].

Respondents living with their care recipient were more likely to report changes to their VPA in either a positive or negative direction. The reason for this inconsistent finding is unclear. It may well be that informal caregivers are exercising more because they have additional time to do so. It is possible that increased VPA is due to increased caregiving commitments due to the lack of paid support during the pandemic, as caregiver burden (as measured by the CBI) also was associated with a reported increase in VPA. It is also possible that respondents who had been diagnosed with COVID-19 had a decrease in cardiopulmonary function and as a result perceived their MPA as VPA. It also is possible, although not assessed in this study, that for some respondents participation in VPA declined due to efforts to stem the pandemic that inhibited their ability to be physically active (e.g.., sheltering at home, closing of gyms and health clubs).

Respondents living with their care recipients were also more likely to report an increase in SB compared to those not living with the person for whom they were providing care. Caregiving burden may impact the health-promoting behaviors of caregivers [43], and prior research suggests that co-residence of caregivers and care recipients may increase CB to some extent [44]. The increase in SB could also be due to spending more time with the care recipient due to less outside caregiving support and this could involve sedentary activities such as watching TV and talking or decreases in PA.

More than half (53%) of respondents reported that they had been diagnosed with COVID-19, which was associated with reported increases in MPA and VPA, and weekend ST during the pandemic. It is possible that the COVID-19 diagnosis served as a cue to action and motivated respondents who had been diagnosed to improve their PA behaviors or they perceived their activity as more strenuous than before due to their illness as discussed previously.

A limitation of the study is that COVID-19 diagnosis is based on self-reported data and that the date of diagnosis and severity of symptoms is not known. It should be noted that the sample reported a substantially higher cumulative incidence (53%) of having COVID-19 than the public, especially considering the data were collected during the early months of the pandemic (June 2020). It may be that caregivers who had been diagnosed with COVID-19 were more interested in participating in the study than those who had not had COVID-19. The sample was 69% male and prior research indicates that women are more likely to be informal caregivers than men [45]. Furthermore, some research conducted pre-pandemic has found that the majority of MTurk workers are women [46]: however, recent research revealed that during the pandemic more males than female participate in social science HITs [47]. These factors limit the generalizability of study findings. Other study limitations include the cross-sectional study design, reliance on self-report measures that were developed for this study, use of a convenience sample of MTurk respondents, and limiting the sample to individuals with internet access. Some research does suggest that MTurk respondents are younger, have lower incomes, and are less likely to be Black than average Americans [48, 49] Nonetheless, research indicates that online convenience samples tend to provide valid results for research [48, 50]. Lastly, the data were collected in June 2020, the early stages of the pandemic, and PA, SB, and ST may have changed since the survey was conducted due to changes in pandemic severity and season [51].

The study has several strengths. It is novel in that it is one of the first studies to explore changes in the health behaviors of informal caregivers, who serve a vital role in the US healthcare system due to the pandemic. Additionally, the sample is relatively large. Moreover, we surveyed several different forms of the behaviors of interest (i.e., three measures of SB, two measures of PA) to allow for a comprehensive exploration among information caregivers of how the pandemic may influence health behaviors related to PA and ST. Future research should focus on uncovering the reasons for the seemingly contradictory findings that living with a care recipient was associated with both increased and decreased likelihood of VPA. It may well be that there are underlying subgroups that could help determine which respondents are more likely to report increased or decreased VPA and uncovering these subgroups may help inform interventions to promote health behaviors in the subgroups that reported decreased healthy behaviors. For example, it could be it could be the type of illness/condition their care giving recipient has that impacts VPA, caregiving for people who need help showering and toileting is different than caregiving for people who do not this type of assistance. These findings may also be an example of the Table 2 Fallacy [52], so future research in this area should consider using a causal inference approach to determine if these results are statistical or causal associations. Future research could also examine longitudinal changes in caregiving to determine if changes in supports and/or increases in caregiving are associated with changes in in informal caregivers’ health behaviors.

## Conclusion and implications

The majority of informal caregivers in the current study perceived that their SB, weekday ST, and weekend ST increased during the pandemic. The findings of the current study indicate a need to intervene on these behaviors among informal caregivers for older adults to return to pre-pandemic behaviors for those who reported an increase in negative health behaviors or a decrease in health promoting behaviors. In addition, intervention efforts could also be implemented to reinforce positive changes in behaviors. Special efforts should be given to informal caregivers who reside with their care recipient. Their health behaviors could be assessed at their medical appointments or when they accompany the care recipient to appointments. Assessments also could be done in person, via telehealth appointment, or online. Interventions that can be delivered remotely should be explored due to time constraints associated with caregiving.

## Data Availability

The dataset analyzed for this current study are available from the corresponding author upon reasonable request.
